# Messenger RNA Gene Expression Screening of VIP and PACAP Neuropeptides and Their Endogenous Receptors in Ruminants

**DOI:** 10.3390/biology11101512

**Published:** 2022-10-15

**Authors:** Emma Hawley, Kafi Mia, Mustapha Yusuf, Kendall C. Swanson, Curt Doetkott, Glenn P. Dorsam

**Affiliations:** 1 Department of Microbiological Sciences, North Dakota State University, Fargo, ND 58102, USA; 2 Department of Animal Sciences, North Dakota State University, Fargo, ND 58102, USA; 3 Information Technology Services, North Dakota State University, Fargo, ND 58102, USA

**Keywords:** PAC1, PACAP, ruminant, steer, wether, VIP, VPAC1, VPAC2

## Abstract

**Simple Summary:**

This study aimed to measure the neuropeptides, vasoactive intestinal peptide (VIP) and pituitary adenylate cyclase activating polypeptide (PACAP), and their receptors, termed VPAC1, VPAC2 and PAC1 in ruminants. To date, we are unaware of any reported quantitative polymerase chain reaction (qPCR) measurements for these genes in either sheep (weathers) or cows (steers). To this end, we isolated total RNA from 15 different tissues from both wethers and steers and performed qPCR measurements. These data revealed expression for VIP and PACAP in the brain and intestines of both ruminant species, while VPAC1 and PAC1 receptors were detected in the brain, throughout the intestines (e.g., duodenum, jejunum, ilium, and colon), metabolically relevant organs (e.g., liver, kidney, and fat), and spleen (a primary immune organ). In contrast, VPAC2 was not detected in wethers, and only detected in spleen and omasum (muscular third stomach) in steers. Collectively, these data reveal for the first-time tissue expression profiles for the VIP and PACAP ligands and their receptors in ruminants that will provide researchers a better understanding of their biological activities in these animals.

**Abstract:**

Vasoactive Intestinal Peptide (VIP) and Pituitary Adenylate-Cyclase-Activating Peptide (PACAP) are anti-inflammatory neuropeptides that play important roles in human and rodent gut microbiota homeostasis and host immunity. Pharmacologically regulating these neuropeptides is expected to have significant health and feed efficiency benefits for agriculturally relevant animals. However, their expression profile in ruminant tissues is not well characterized. To this end, we screened for VIP and PACAP neuropeptides and their endogenous GPCRs using 15 different tissues from wethers and steers by RT-qPCR. Our results revealed relatively similar expression profiles for both VIP and PACAP neuropeptide ligands in the brain and intestinal tissue of both species. In contrast, the tissue expression profiles for VPAC1, VPAC2, and PAC1 were more widespread and disparate, with VPAC1 being the most diversely expressed receptor with mRNA detection in the brain and throughout the gastrointestinal tract. These data are an important first step to allow for future investigations regarding the VIP and PACAP signaling pathways in livestock ruminant species.

## 1. Introduction

Livestock animals are utilized for food, labor, and other commodities worldwide [1,2]. Prices of grain and other feed ingredients have increased over the past 30 years [3]. Prior to animal harvest, producers often feed high-concentrate diets to maximize animal weight resulting in increased production of volatile fatty acids (VFA) within the rumen [4,5]. Volatile fatty acids are energy-rich metabolites produced by the gut microbiota that are the primary dietary energy source for ruminants [6]. However, feeding high concentrate diets can also result in excess VFA production, which lowers ruminal pH and induces subacute ruminal acidosis (SARA) along with a proinflammatory tone that compromises digestibility, feed intake, and milk production thus reducing profits [7]. Therefore, there is a need to further explore management options for decreasing ruminal SARA and improving the efficiency of nutrient utilization by manipulating the signaling pathways of anti-inflammatory neuropeptides known to be expressed within the gastrointestinal tract of ruminants.

Vasoactive intestinal peptide (VIP) and/or pituitary adenylate cyclase activating polypeptide (PACAP) regulate circadian rhythm, thermogenesis, immunity, and metabolism [8,9,10,11,12]. Both neuropeptides belong to the secretin superfamily, share more than 68% amino acid identity to each other, bind similar G protein-coupled receptors (GPCR), termed VIP/pituitary adenylyl cyclase activating polypeptide (VPAC) receptor 1, VPAC2 and PAC1, and are identical at the amino acid level in humans, mice, and ruminant species, like sheep (wethers) and cows (Steers) [13,14,15]. Within the mucosa-associated lymphoid tissue of the gastrointestinal tract (gut), both peptides are delivered to the gut and profoundly influence physiological processes, including gastric acid secretion by the stomach, water/ion absorption in the large intestine, peristalsis by inhibiting smooth muscle contraction, local blood flow, exocrine secretions from the pancreas, cell migration, proliferation and mucus secretion by Goblet cells [16,17,18,19,20,21] and reviewed in [22,23]. With respect to inflammatory gut disorders, such as inflammatory bowel diseases (IBD), studies have revealed elevated PACAP mRNA in dextran sulfate sodium (DSS)-induced inflammatory colitis, and genetically deficient PACAP mice exhibited reduced levels of proinflammatory cytokines throughout the gut [24,25]. Consistently, exogenously added PACAP can ameliorate acute ileitis pathology as well as induce higher survival rates [26]. The most recent report demonstrated that peritoneal injections of VIP can alleviate DSS-induced inflammatory colitis by inhibiting pro-inflammatory cytokine expression in mice [27]. Additionally, VIP has also been shown to stimulate the release of cholecystokinin (CKK) from the small intestine and digestive enzymes from the pancreas [28], making it a potential candidate for improving starch digestion in ruminants, which is important because these animals have limitations in their ability to digest starch in the small intestines [29,30]. Importantly, VIP has been shown to enhance nutrient absorption in mice [31]. Curiously, only PACAP deficient mice showed an increased risk for colorectal cancer incidence during DSS-induced inflammation [32]. Lastly, within the last five years, researchers have observed that deficiency in either VIP or PACAP results in significant gut microbiota dysbiosis [33,34] and VPAC1 may be a principle driving force mediating these homeostatic effects on the gut microbiota ecology in mice [35].

We expect that capitalizing on the anti-inflammatory, cytoprotective and metabolic effects of these neuropeptides might provide a novel strategy to improve the health of ruminants fed a high-concentrate diet by simultaneously blunting SARA, while improving feed efficiency. However, there is a paucity of expression studies for the VIP/PACAP signaling axes in ruminants. Therefore, the first phase of this research is to establish an mRNA expression profile defining the distribution of VIP and PACAP ligands and their cognate GPCRs in ruminants that will help predict tissue-specific effects.

In the present study, we measured relative mRNA expression of VIP, PACAP, and their endogenous 3 GPCRs across 15 different tissues in wethers and steers. Our results demonstrate a relatively consistent expression profile for both ligands in the gut and brain, with a more varied expression profile for their receptors between these two-ruminant species. This neuropeptide expression profile in ruminants is consistent with that reported in humans and mice. Understanding the mRNA expression tissue distribution in ruminants is a necessary first step in determining whether manipulating these neuropeptide pathways could provide farmers with a superior animal management strategy against SARA when feeding ruminants a high-concentrate diet.

## 2. Materials and Methods

### 2.1. Tissue Harvest

All animal research was approved by the North Dakota State University Institutional Animal Care and Use Committee (IACUC) under the beef cattle herd IACUC protocol number A20074. Animals were slaughtered in the North Dakota State University Meat Laboratory, which is inspected by the United States Department of Agriculture (USDA). Animals were not slaughtered specifically for this study. All tissues used for this study were collected post-slaughter from the Meats Department at NDSU (Abattoir). End-point analyses conducted were approved by the institutional biosafety committee protocol number B19027.Tissues were collected from steers predominately of Angus and Simmental breeding that were between 455 and 621 days old and weighed between 501 to 540 kg, and wethers predominately of Dorset breeding were between 252 and 276 days old and weighed 72 to 88 kg. Both steers and heifers were fed concentrate-based finishing diets and were euthanized via captive bolt and exsanguination. Tissues collected were: (1) brain, (2) duodenum, (3) jejunum, (4) ileum, (5) cecum, (6) colon, (7) rumen, (8) reticulum, (9) omasum, (10) abomasum, (11) muscle, (12) fat (omental), (13) liver, (14) spleen, and (15) kidney, and frozen at −80 °C until assayed.

### 2.2. RNA Extraction and cDNA Synthesis

**Figure 1:**
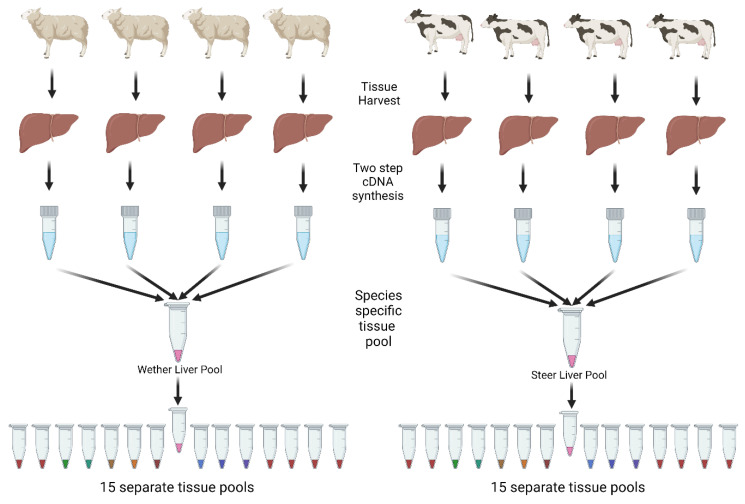
cDNA pooling strategy used for species-specific tissues prior to qPCR screening. A total of 15 tissues were harvested from 4 steers and 4 wethers. RNA was extracted from all tissues and used to create a cDNA library. There was a total of 4 biological replicates for each tissue in both species and these cDNAs were pooled into a single sample for qPCR screening of VIP, VPAC1, VPAC2, PACAP, PAC1, and reference genes.

The minimum information for publication of quantitative real-time PCR experiments (MIQE) was adhered to for the RT-qPCR analysis wherever possible (Table A1) [36]. RNA was extracted using the Qiagen Universal Mini-RNA Extraction Kit (Catalogue #73404, Germantown, MD, USA). RNA quantification and purity assessment were determined by spectroscopy with a NanoDrop™ one-C (Waltham, MA, USA) by measuring absorbance at 260 and 280 nm, with total RNA average yields ranging from 7 to 92 µg and 260/280 nm ratios falling between 1.9 and 2.3 (Table A2). RNA integrity was measured using a Qubit 4 fluorometer to measure the extent of degraded total RNA (scale is 1–10 with 10 representing the highest intact RNA) and samples with an IQ score ranging from 6.4 to 10 were considered acceptable (ThermoFischer Scientific, Waltham, MA, USA, 2018). All total RNA samples were treated with DNase I and 10xReaction Buffer with MgCl_2_ (Thermo Fischer Scientific: EN0521; B42) for 30 min at 37 °C, followed by a 10-min 70 °C deactivation step with a final concentration of 5 mM EDTA. A range of 0.62 to 0.8 µg of total RNA was used for each sample. cDNA synthesis was conducted using the Solis BioDyne FIREScript RT cDNA synthesis kit (#06-15-00050, Tartu, Estonia). Briefly, cDNA reactions contained final concentrations of FIREScript RT (10 U/µL), RiboGrip^TM^ RNase inhibitor (1 U/µL), dNTP MIX (500 µM of each), oligo dT primers (2.5 µM), random primers (2.5 µM), and 1x RT reaction buffer with dithiothreitol (DTT) with final concentrations of 50 mM Tris-HCl pH 8.3, 50 mM KCl, 3 mM MgCl2, and 10 mM DTT. Reverse transcriptase reactions were incubated at 25 °C for 5 to 10 min, 37 to 60 °C for 15–30 min, and 85 °C for 5 min to deactivate the reverse transcriptase enzyme. A total of 4 biological replicates (*n* = 4) were pooled for each tissue cDNA group, except for fat and brain, which contained 3 replicates (*n* = 3). Pooled biological replicates of tissue samples (Figure 1) were diluted 1:10 with water to limit PCR inhibitors and frozen until used for RT-qPCR analysis.

### 2.3. qPCR

**Figure A1:**
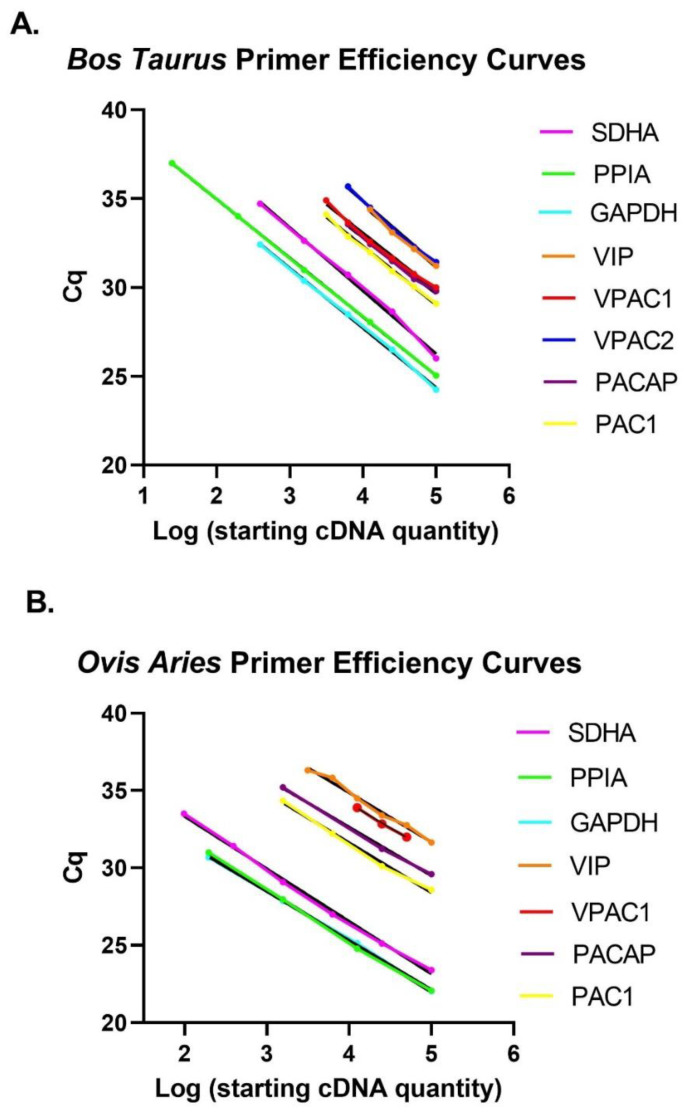
Primer Efficiency Curves. Curves made using (**A**)**.** *Bos Taurus* and (**B**). *Ovis Aries*. Efficiencies are between 91–108%.

**Figure A2:**
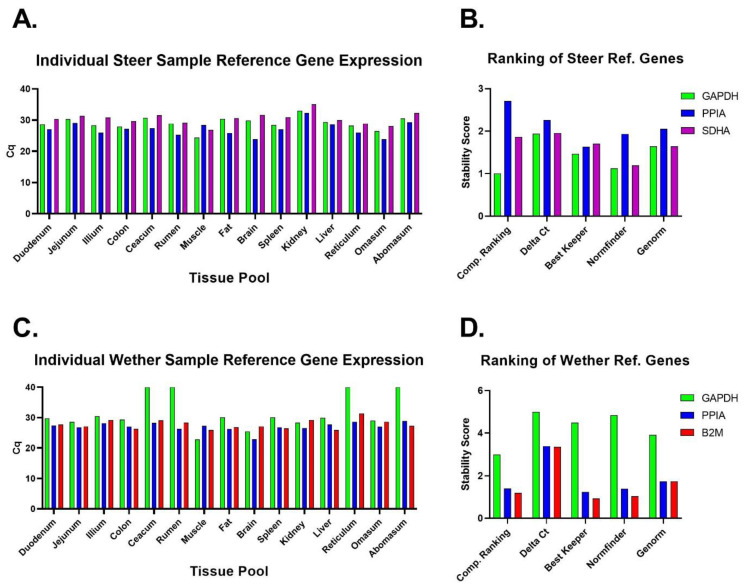
Reference Gene Stability Assessment Across Steer and Wether Samples. Average of technical replicates and stability score ranking of reference genes GAPDH, PPIA, B2M and SDHA in (**A**,**B**). Steers and (**C**,**D**). Wethers.

**Figure A3:**
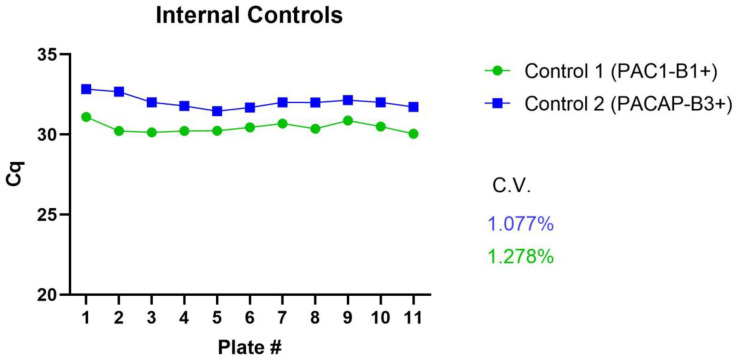
Coefficient of Variation Between Plates on Separate qPCR Runs. Inter-assay plate control trends with an associated coefficient of variation (C.V.). Data points represent the mean of technical replicates for each plate. Plate # refers to the number of independent qPCR plates performed for this analysis.

Quantitative PCR (qPCR) was conducted using the BioRad CFX96 thermocycler (Hercules, CA, USA) and 96-well plates were prepared according to the manufacturer’s protocol. Briefly, reactions contained final concentrations of 1X HOT FIREPol Evagreen qPCR Supermix (08-36-0000S) and 200 nmols of forward and reverse primers specific for the gene of interest (Table 1), with 5 µL of 1/10 diluted cDNA samples and volumes brought to 20 µL final volume with nuclease-free water. Amplification parameters were 95 °C ^(12 min)^ hot start + [95 °C ^(15 s)^ denaturation + 60 °C ^(30 s)^ annealing] × 40 cycles. Melt curves with 5-s intervals between 65 and 95 °C were utilized for all PCR experiments, and only those primer pairs that showed a single amplicon PCR product were used. Primer efficiencies for all reactions were between 91 and 108% as suggested by MIQE (Table 1 and Figure A1). The values of Cq were measured using the regression determination method of BioRad Manager 3.1 software (updated 6 June 2021, Hercules, CA, USA). Cq values less than 36 were considered above the limit of detection and used to calculate relative quantification based on the 2^−^^ΔΔCq^ method [37], and qPCR reactions were performed in duplicate. Three reference genes were optimized for each species (Figure A2) and the two most stable reference genes were used for normalization based on Genorm calculation [38]. The percent coefficient of variation [(standard deviation/mean) × 100] of intraassay controls was below 1.3% (Figure A3). No reverse transcriptase (NRT) and no template controls (NTC) were assessed for each sample to determine the extent of genomic DNA contamination. All NRT and NTC reactions had fluorescence signals in all RT+ reactions of at least 92% from the mRNA pool based on the following formula: RT+ fluorescence = 100% − [2(^−(Cq NRT or NTC)/(−2^(−Cq RT+)^) × 100] [37].

### 2.4. Data Analysis

Relative quantification for mRNA expression levels [37] (BioRadLifeScience, Hercules, CA, USA, 2019) were graphed by arbitrarily setting VIP brain levels from each species to 1. Two technical replicates of tissue samples were performed from pooled cDNA and averages +/− SD are presented in bar graph form.

## 3. Results

### 3.1. VIP and PACAP Ligands Share a Similar mRNA Tissue Expression Profile between the Gut and Brain

**Figure 2:**
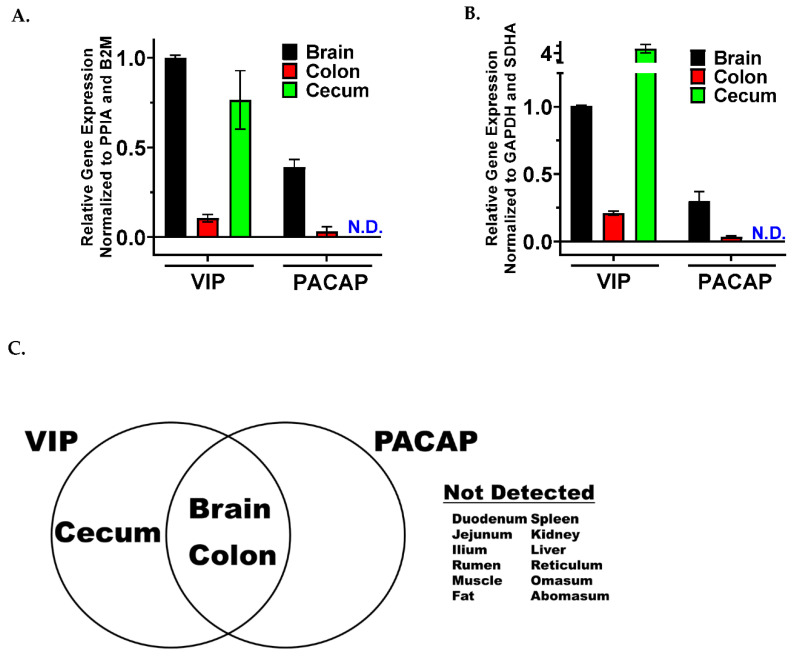
Similar relative mRNA profiles for VIP and PACAP ligands. Data is presented in bar graph form with technical duplicate means +/− SD of pooled cDNA (*n* = 3 − 4) for indicated tissues measured in (**A**). wethers or (**B**). steers. N.D. indicates no detection. (**C**). Venn diagram representing tissue expression for ligands with overlap indicating identical expression profiles between species.

Investigations into the VIP/PACAP signaling axis have been performed in rodents and humans for the past 30 years [15,40], and both neuropeptides have been suggested to be signaling members in the “gut-brain” axis [41]. In contrast, there is a paucity of published research on these neuropeptides and receptors in ruminant species regarding their gene expression profiles. Therefore, we set out to measure the relative mRNA expression levels for both neuropeptides, VIP and PACAP, and their cognate GPCRs, VPAC1, VPAC2 and PAC1. To this end, we collected 15 different tissues from wethers and steers, extracted total RNA, synthesized cDNA, and pooled cDNA biological replicates (*n* = 3 − 4) from each of the 15 tissue types per species (Figure 1). VIP and PACAP mRNA were detected in only 3 of the 15 tissues studied (Figure 2C). Both peptides were detected in the brain (highest relative mRNA levels for PACAP and VIP in wethers) and colon (lowest relative mRNA levels) for both species (Figure 2A,B). In addition, VIP was expressed in the cecum, which showed higher relative expression in steers compared to wethers. Unexpectedly, the variability of VIP mRNA levels for cecum in wethers was unusually high and we cannot satisfactorily explain such high standard deviation. However, since VIP expression in cecum was the only sample out of 210 qPCR reactions performed in duplicate (15 tissues analyzed for 7 genes in 2 species) exhibiting large intraassay variability amounting to ≤1% of the qPCR reactions tested, and that VIP also was detected in cecum from steers, we reasoned that these nonideal results are still well within normal experimental error expectations. Collectively, we conclude that VIP and PACAP share a common, yet narrow, mRNA expression profile focused within the brain and gut of wethers and steers.

### 3.2. VIP and PACAP GPCRs Have a More Varied and Widespread mRNA Tissue Expression Profile Compared to Their Ligands

**Figure 3:**
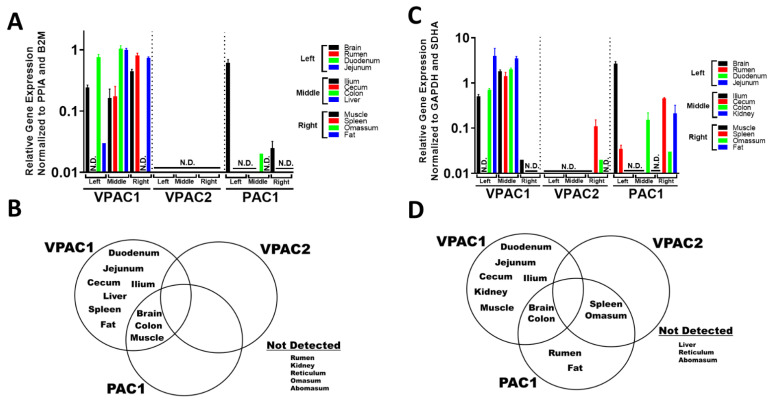
Differential relative mRNA profiles for VIP and PACAP receptors. Data is presented in bar graph form with technical duplicate means +/− SD of pooled cDNA (*n* = 3 − 4) for indicated tissues measured in (**A**). wethers or (**C**)**.** (**C**,**D**). Venn diagrams representing tissue expression of VIP/PACAP receptors with overlap indicating identical expression profiles between (**B**). wethers and (**D**). steers.

Next, we focused on the mRNA expression levels for the 3 VIP/PACAP GPCRs by using pooled cDNA samples as previously described and repeated RT-qPCR. In contrast to the VIP/PACAP ligands, the combined VPAC1, VPAC2, and PAC1 mRNA profiles exhibited a more widespread expression profile with mRNA detection in 10 and 12 tissues out of 15 for wethers and steers, respectively. In tissues that exhibited mRNA expression of at least 1 of the 3 VIP/PACAP GPCRs, VPAC1 was expressed in all 10 tissues in wethers, and in 8 of the 12 tissues in steers, respectively (Figure 3C,D). VPAC1 exhibited the highest average expression levels across multiple tissues in both species (Figure 3A,B). VPAC2 mRNA expression was the most sparsely detected GPCR, with no detection in wethers and only moderate to low detection in spleen and omasum of steers (Figure 3A,B). PAC1 was expressed in an intermediate number of tissues (Figure 3C,D). In wethers, PAC1 was co-expressed with VPAC1 in colon, brain and muscle, while in steers it shared expression with VPAC1 in colon and brain, but spleen and omasum with VPAC2 (Figure 3C,D). Only PAC1 brain mRNA levels were statistically higher than VPAC1 brain mRNA levels in wethers and steers (Figure 3A,B). Tissue expression rankings are summarized in Table 2. This mRNA expression profile between ligand and GPCRs is consistent with the high degree of primary amino acid sequence identity (Table 3) for VIP and PACAP (100%), whereas their GPCRs exhibit far more divergence in amino acid sequence (78.9–90.7%). These observations demonstrate a strikingly similar mRNA expression profile for the VIP and PACAP ligands in tissues of wethers and steers. In stark contrast, the three VIP/PACAP GPCRs exhibited a far wider tissue distribution, with similarities and differences in wethers and steers, that could dictate their biological influences in these ruminant species.

## 4. Discussion

This study aimed to screen mRNA expression of VIP, PACAP, and their endogenous GPCRs in 15 different tissues from two different ruminant species, wethers, and steers. To our knowledge, this study is the first screening attempt to measure mRNA expression levels of the VIP and PACAP signaling axes in ruminants. This analysis adhered to the MIQE recommendations and generated useful tools for future ruminant research with the successful generation of 9 qPCR optimized primer pairs, some of which are suitable for analyzing their target cDNA products in both species.

VIP and PACAP ligands were readily detected in the gut and brain of wethers and steers and support the rodent and human research demonstrating that these peptides are signaling members of the gut-brain axis [41]. Wethers and steers expressed ligand genes sparsely with detection in only 3 of 15 tissues but exhibited 100% tissue expression agreement between species for VIP (3/3) and PACAP (2/2), respectively. In contrast, wethers and steers expressed their GPCR genes more broadly with at least one receptor gene detected in 13 of 15 tissues but showed less tissue expression agreement between both ruminant species with VPAC1 showing 64% agreement (7/11), VPAC2 showing no agreement (0/2), and PAC1 showing 29% agreement (2/7), respectively. The observed brain and colon expression for VIP and PACAP in the two ruminant species tested supports their classification as neuropeptides, as this central and peripheral (enteric) nervous system expression profile is also seen in other animals, including humans, rodents, cats, pigs and ferrets [23,42]. In rodents, VIP displays the highest mRNA expression in the large intestine and cortex/frontal lobe regions [43,44], and humans express VIP mostly in the appendix (cecum), colon, small intestine, and brain [45]. VIP was also detected in the cecum of steers and wethers, which could be related to the necessity for maintaining cecal size and microbiota homeostasis [18,33,34,46]. Similarly, PACAP was detected in the brain and colon of both ruminant species, congruent with its high mRNA expression in the central nervous system and colon of humans and mice [18,34,43,45]. Curiously, there were only three tissues that expressed either VIP or PACAP ligands in wethers and steers, which at first glance may not appear consistent with their expression profiles in rodents and humans [23,42]. However, in addition to these neuropeptides being expressed in the mammalian nervous system, their expression has also been observed in cells of the innate and adaptive immune system [47]. One potential explanation for the sparsity in tissue expression for VIP and PACAP in the ruminant species tested could be due to their neuronal and/or immune cell expression profiles observed in smaller animals, like rodents. For example, tissue innervation by VIP^+^ or PACAP^+^ neurons and/or resident immune cells in larger ruminant animals might be diluted out by the larger organ size. This would be consistent with VIP and PACAP expression observed in nerve fibers and/or immune cells in numerous mammals, including sheep, that measured protein expression by immunohistochemistry [23]. Future experiments are warranted to investigate gene expression of the VIP/PACAP signaling axes by analyzing neuron innervation and tissue-specific immune cells directly by immunohistochemical and flow cytometry assays to better delineate VIP/PACAP expression in these larger ruminant animals.

Our present study shows that, apart from VPAC2 in wethers, the GPCR mRNA were detected throughout the gut (e.g., duodenum through the colon), brain, metabolically relevant organs (e.g., liver, kidney, and fat), and spleen (a primary immune organ) in steers and wethers. Consistent with this profile in ruminants, VPAC1 has been reported to be the most widely expressed VIP/PACAP GPCR, with high expression focused in the gut, brain, fat, liver, and spleen of mice and humans [43,45,48]. Interestingly, VPAC1 mRNA being the most widespread and robustly expressed of the of the 3 GPCRs was absent from rumen, with only PAC1 mRNA found in the rumen of steers, and PAC1 and VPAC2 weakly detected in the omasum. The detection of PAC1 in steer rumen implies that PACAP may have a specific effect on ruminal biology. Future research is needed to explore this possibility.

There were, however, some differences in the detectability of receptor expression in steers and wethers. First, there was no detectable expression of VPAC1 in the spleen or fat pads of steers, suggesting a species difference from mice [44,48,49], humans [50], and wethers (present study). Second, muscle tissue showed VPAC1 expression in both ruminants tested, whereas this tissue did not show the same expression profile in mice or humans. This discrepancy may again be due to VPAC1^+^ nerves innervating muscle tissue, which may not reach detectable levels by qPCR [51]. Third, we measured high VPAC2 expression in steer muscle, but VPAC2 expression was undetectable in all tissues tested from wethers. A lack of VPAC2 expression in wethers is supported by a previous report demonstrating very low reads per kilobase million (RPKM) for VPAC2 in sheep tissue [52]. Indeed, VPAC2 has been observed to be upregulated in immune cells during inflammation and could also be a contributing factor for why we observed very little detectible VPAC2 expression in the current study [53]. Lastly, wethers showed VPAC1 expression in the spleen consistent with rodents and humans [44,54], but in contrast, steers showed VPAC2 and PAC1 expression prompting questions regarding their role in the immunological functions of these receptors between wethers and steers.

This study has many notable limitations. The pooling of cDNA samples from four biological replicates was done to reduce the total number of analyses, thereby limiting the statistical power of this initial screening study. Future research is necessary to validate these mRNA expression profiles using biological replicates, rather than pooled technical replicates. Also, the expression profiles collected are only at the mRNA level and may not represent accurate protein tissue distribution. Analyses like immunohistochemistry and flow cytometry will be invaluable in confirming not only protein expression profiles but also allow for identification of specific cell sources contributing to their expression, especially in heterogeneous organs like brain and intestines. Lastly, primers spanning exons 10 and 11 of the PAC1 gene were used for this study and based on present knowledge of PAC1 splice variants in human, mouse and zebra fish, the current study is likely measuring all PAC1 mRNA species [55,56]. Additional research is required to investigate which PAC1 transcripts are expressed in the relevant tissues identified in this study.

In conclusion, these mRNA expression data in ruminants are consistent with the categorization of VIP/PACAP as neurotransmitters expressed in the central (e.g., brain) and peripheral (e.g., colon and cecum) nervous systems in other mammalian species. Likewise, ruminant VIP/PACAP GPCR expression is also predominantly represented within the brain and the gut, especially VPAC1, that mediates the physiological actions of these evolutionarily conserved peptides. Based on this mRNA screening study, it now seems reasonable to explore the possibilities for capitalizing on the VIP/PACAP signaling pathways to improve gut and immune function in ruminants during high carbohydrate finishing diets.

## Figures and Tables

**Table 1:** Primer Specifics. All primers were designed using NCBI Primer Design [39]. Each row denotes a specific gene, optimized species, F&R primer sequences (5′ > 3′), targeted sequence accession #, product length in base pairs (BP), percent efficiency, slope, R^2^, MT in RT^+^ samples, and exon boundary information. B = *Bos Taurus (Steer)*, O = *Ovis Aries (Wether)*.

Gene	Species	Primer Pair Sequence (5′ > 3′)	Target Sequence Accession #	Product Length (bps)	Efficiency	Slope	R^2^	Product MT °C	Exon–Exon Boundary (nt)
VIP	B	F: CCACTCAGATGCTGTCTTCACT R: TTCACTGCTTCGCTTTCCATTTAG	NM_173970.3	103	94.1%	−3.47	0.99	80.0	5–6 (642/643)
VIP	O	F: CACTGACAACTACACACGCC R: GACTCTCCTTCGCTGCTTCTC	NM_001126368.1	93	105.6%	−3.1	0.99	79.0	4–5 (467/468)
PACAP	B	F: TGTACGACGAGGACGGAAAC R: GTGGGCGACATCTCTTTCCT	NM_001046555.1	131	107.4%	−3.12	0.99	90.5	2–3 (242/243)
	O		NM_001009776.1	131	108.6%	−3.13	0.99	90.5	N/A
VPAC1	B	F: ATCCTTGCCTCCATCTTGGTG R: GCTGTCACTCTTCCCGACAT	NM_001081607.1	99	103.1%	−3.25	0.99	81.5	9–10 (1029/1030)
	O		XM_042235879.1	99	107.5%	−3.15	0.96	81.5	N/A
VPAC2	B	F: CATCCGCATCTCCTCCAAGTA R: TCTGCACCTCGCTGTTGA	NM_001206781.1	107	90.9%	−3.56	0.99	84.5	12–13 (1287/1288)
PAC1	B	F: ATCATCATTGGCTGGGGGAC R: ATGATGCCGATGAAGAGCACA	NM_175715.2	176	101.4%	−3.29	0.99	85.5	10–11 (1371/1372)
	O		XM_027968637.2	176	104.3%	−3.22	0.99	85.5	N/A
GAPDH	B	F: TCGGAGTGAACGGATTCGGC R: TGATGACGAGCTTCCCGTTC	NM_001034034.2	192	98.5%	−3.36	0.99	80.5	2–3 (94/95)
	O		NM_001190390.1	192	106.4%	−3.18	0.99	80.5	2–3 (62/63)
PPIA	B	F: GCCAAGACTGAGTGGTTGGAT R: TTGCTGGTCTTGCCATTCCT	NM_178320.2	113	100.6%	−3.31	1.00	84.5	4–5 (373/374)
	O		NM_001308578.1	113	100.3%	−3.31	0.99	84.5	4–5 (363/364)
SDHA	B	F: TCCTGCAGACCCGGAGATAA R: TCTGCATGTTGAGTCGCAGT	NM_174178.2	130	91.2%	−3.55	0.99	81.0	10–11 (1446/1447)
B2M	O	F: CTGCTGCAAGGATGGCTGTCT R: GGACCTCTGGAATACGCTGGAT	NM_001009284.2	93	96.9%	−3.39	0.99	87.5	1–2 (79/80)

**Table 2:** Relative mRNA Expression Ranking in Ruminant Tissues. Relative expression rank: High—++, Intermediate—+, Low—−/+, and not detected—ND.

	Wether	Steer	Wether	Steer	Wether	Steer	Wether	Steer	Wether	Steer
Brain	++	+	+	−/+	++	+	ND	ND	++	++
Rumen	ND	ND	ND	ND	ND	ND	ND	ND	ND	−/+
Duodenum	ND	ND	ND	ND	+	+	ND	ND	ND	ND
Jejunum	ND	ND	ND	ND	−/+	++	ND	ND	ND	ND
Ilium	ND	ND	ND	ND	+	++	ND	ND	ND	ND
Cecum	++	++	ND	ND	+	++	ND	ND	ND	ND
Colon	−/+	−/+	−/+	−/+	++	++	ND	ND	−/+	+
Liver	ND	ND	ND	ND	++	++	ND	ND	ND	ND
Muscle	ND	ND	ND	ND	+	−/+	ND	ND	−/+	ND
Spleen	ND	ND	ND	ND	+	ND	ND	+	ND	+
Omasum	ND	ND	ND	ND	ND	ND	ND	−/+	ND	−/+
Fat	ND	ND	ND	ND	+	ND	ND	ND	ND	+
Kidney	ND	ND	ND	ND	ND	ND	ND	ND	ND	ND
Reticulum	ND	ND	ND	ND	ND	ND	ND	ND	ND	ND
Abomasum	ND	ND	ND	ND	ND	ND	ND	ND	ND	ND

**Table 3:** Superfamily Member Identities. Species names, amino acid sequence of VIP and PACAP, amino acid lengths of endogenous receptors, and amino acid percent identify as indicated in *Homo sapiens, Mus musculus, Ovis aries, Bos taurus*. Identity aligned with NCBI multiple alignment tool: Identity = # of similar amino acids/total number of amino acids [15]. * Compositional Bias.

Peptide	AA Sequence or REF #	% Identity to *Homo sapiens*
VIP		
*Homo sapiens*	HSDAVFTDNYTRLRKQMAVKKYLNSILN	100%
*Mus musculus*	HSDAVFTDNYTRLRKQMAVKKYLNSILN	100%
*Bos taurus*	HSDAVFTDNYTRLRKQMAVKKYLNSILN	100%
*Ovis aries*	HSDAVFTDNYTRLRKQMAVKKYLNSILN	100%
	PACAP-38	
*Homo sapiens*	HSDGIFTDSYSRYRKQMAVKKYLAAVLGKRYKQRVKNK	100%
*Mus musculus*	HSDGIFTDSYSRYRKQMAVKKYLAAVLGKRYKQRVKNK	100%
*Bos taurus*	HSDGIFTDSYSRYRKQMAVKKYLAAVLGKRYKQRVKNK	100%
*Ovis aries*	HSDGIFTDSYSRYRKQMAVKKYLAAVLGKRYKQRVKNK	100%
	VPAC1	
*Homo sapiens*	sp|P32241|31-457	100%
*Mus musculus*	sp|P97751|31-459	84.3%
*Bos taurus*	tr|F1MF07|31-457	89.9%
*Ovis aries*	tr|W5NZL6|31-492	78.9%
	VPAC2	
*Homo sapiens*	sp|P41587|24-438	100%
*Mus musculus*	sp|P41588|23-437	87.7%
*Bos taurus*	tr|F1MIT6|25-442	86.3%
*Ovis aries*	* tr|W5PKZ4|20-96, 109-363, 369-424	79.8%
	PAC1	
*Homo sapiens*	sp|P41586|21-468	100%
*Mus musculus*	sp|P70205|21-496	88.4%
*Bos taurus*	sp|Q29627|38-513	90.7%
*Ovis aries*	tr|W5PCC2|21-520	81.8%

## Data Availability

All raw data for this manuscript is available upon request. Please contact glenn.dorsam@ndsu.edu for all inquiries.

## Appendix A

**Table A1:** MIQE Checklist. Descriptions of each parameter of the MIQE Checklist. E = All essential information. D = Desired Information that is submitted if available.

Item to Check	Importance	Checklist	Details
Experimental Design			
Definition of experimental and control groups	E	✓	4 stears 455–621 days old and 4 wethers 252–276 days old.
Number within each group	E	✓	A total of 4 biological replicates were pooled in each tissue cDNA group, except for fat and brain, which only contained 3 replicates.
Assay carried out by core lab or investigator’s lab?	D	✓	Investigator’s lab
Acknowledgement of authors’ contributions	D	✓	K.M, M.Y., and K.C.S performed the sheep and cow husbandry and tissue collection. E.H. and K.M. extracted RNA from tissues and EH performed qPCR experiments. E.H. and G.P.D. wrote the sections of the manuscript. G.P.D calculated fold-changes for the dataset and produced the graphs. K.C.S, C.D., and K.M. provided advice for the experimental design. K.C.S., K.M., C.D., and M.Y. critically edited the manuscript. G.P.D. devised the experimental design, established collaborations with K.C.S., K.M., and M.Y., interpreted all the data, graphed the data, organized the tables, and edited the manuscript.
**Sample**			
Description	E	✓	From both steers & wethers: (1) Brain (2) Duodenum (3) Jejunum (4) Ileum (5) Cecum (6) Colon (7) Rumen (8) Reticulum (9) Omasum (10) Abomasum (11) Muscle (12) Fat (13) Liver (14) Spleen and (15) Kidney.
Volume/mass of sample processed	D		
Microdissection or macrodissection	E	✓	Macrodissection
Processing procedure	E	✓	None
If frozen—how and how quickly?	E	✓	Liquid nitrogen.
If fixed—with what, how quickly?	E	✓	No fixation
Sample storage conditions and duration	E	✓	30 min duration between slaughter to freezing. Samples stored at −80 °C
**NUCLEIC ACID EXTRACTION**			
Procedure and/or instrumentation	E	✓	Column and Phenol/chloroform extraction
Name of kit and details of any modifications	E	✓	Trizol, Qiagen Mini or Universal Mini Kits.
Source of additional reagents used	D	✓	Millipore-Sigma
Details of DNase or RNAse treatment	E	✓	Qiagen Mini (qiashredder column), Qiagen Universal Mini (gDNAse included in kit). DNAse also used prior to cDNA (see below)
Contamination assessment (DNA or RNA)	E	✓	All NRT and NTC reactions for this study were ≤ 8% based on the following formula: RT+ fluorescence = 100% − [2^(−(Cq NRT or NTC)/( −2^(−Cq RT+))^ × 100] [37].
Nucleic acid quantification	E	✓	Spectrophotometer
Instrument and method	E	✓	Nanodrop ND-1000 spectrophotometer
Purity (A260/A280)	D	✓	1.9–2.3
Yield	D	✓	7–92 ug
RNA integrity method/instrument	E	✓	Qubit 4 fluorometer
RIN/RQI or Cq of 3′ and 5′ transcripts	E	✓	IQ 6.4–10
Electrophoresis traces	D		
Inhibition testing (Cq dilutions, spike or other)	E	✓	cDNA was diluted 1/10 with water or 1x TE prior to qPCR.
**Reverse Transcription**			
Complete reaction conditions	E	✓	cDNA reactions containing final concentrations of FIREScript RT (10 U/µL), RiboGripTM RNase Inhibitor (1 U/µL), dNTP MIX (500 µM of each), Oligo dT (2.5 µM) Random primers (2.5 µM) and 1x RT Reaction Buffer with DTT with final concentrations of 50 mM Tris-HCl pH 8.3, 50 mM KCl, 3 mM MgCl2, 10 mM DTT
Amount of RNA and reaction volume	E	✓	620–800 ng in 20 µL volume
Priming oligonucleotide (if using GSP) and concentration	E	✓	100 µM Oligo (dT) and 100 µM Random primers
Reverse transcriptase and concentration	E	✓	FIREScript RT (200 U/µl)
Temperature and time	E	✓	Reverse transcriptase reactions were incubated at 25 °C for 5–10 min, 37–60 °C for 15–30 min and 85 °C for 5 min to deactivate the enzyme.
Manufacturer of reagents and catalogue numbers	D	✓	Solis BioDyne FIREScript RT cDNA synthesis kit (#06-15-00050).
Cqs with and without RT	D	✓	Water added to reverse transcriptase (RT) negative control
Storage conditions of cDNA	D	✓	−80 °C for up to 1 year
**qPCR Target Information**			
If multiplex, efficiency and LOD of each assay.	E	✓	Not Multiplex
Sequence accession number	E	✓	See Table 1.
Location of amplicon	D	✓	See Table 1.
Amplicon length	E	✓	See Table 1.
In silico specificity screen (BLAST, etc.)	E	✓	NCBI BLAST and IDT Primer Quest
Pseudogenes, retropseudogenes or other homologs?	D		
Sequence alignment	D		
Secondary structure analysis of amplicon	D		
Location of each primer by exon or intron (if applicable)	E	✓	See Table 1.
What splice variants are targeted?	E	✓	See Table 1.
**qPCR Oligonucleotides**			
Primer sequences	E	✓	See Table 1.
RTPrimerDB Identification Number	D		
Probe sequences	D		
Location and identity of any modifications	E	✓	See Table 1.
Manufacturer of oligonucleotides	D	✓	Solis BioDyne FIREScript RT cDNA synthesis kit (#06-15-00050).
Purification method	D		
**qPCR Protocol**			
Complete reaction conditions	E	✓	Amplification parameters were: 95 °C ^(12 min)^ hot start + [ 95 °C ^(15 s)^ denaturation + 60 °C ^(30 s)^ annealing] × 40 cycles. Melt curves with 5-second intervals between 65–95 °C followed all PCR experiments, and only those primer pairs that showed a single amplicon PCR product were used for this study.
Reaction volume and amount of cDNA/DNA	E	✓	20 µL. At least 93% cDNA
Primer, (probe), Mg++ and dNTP concentrations	E	✓	Solis BioDyne FIREScript RT cDNA synthesis kit (#06-15-00050).
Polymerase identity and concentration	E	✓	Solis BioDyne FIREScript RT cDNA synthesis kit (#06-15-00050).
Buffer/kit identity and manufacturer	E	✓	Solis BioDyne FIREScript RT cDNA synthesis kit (#06-15-00050).
Exact chemical constitution of the buffer	D	✓	Solis BioDyne FIREScript RT cDNA synthesis kit (#06-15-00050).
Additives (SYBR Green I, DMSO, etc.)	E	✓	Solis BioDyne FIREScript RT cDNA synthesis kit (#06-15-00050).
Manufacturer of plates/tubes and catalog number	D	✓	Applied Biosystems Catalogue # 4346907
Complete thermocycling parameters	E	✓	Amplification parameters were: 95 °C ^(12 min)^ hot start + [ 95 °C ^(15 s)^ denaturation + 60 °C ^(30 s)^ annealing] × 40 cycles. Melt curves with 5-second intervals between 65–95 °C followed all PCR experiments, and only those primer pairs that showed a single amplicon PCR product were used for this study.
Reaction setup (manual/robotic)	D	✓	Manual
Manufacturer of qPCR instrument	E	✓	Bio-Rad CFX96
**qPCR Validation**			
Evidence of optimisation (from gradients)	D		
Specificity (gel, sequence, melt, or digest)	E	✓	Single Melting Peak
For SYBR Green I, Cq of the NTC	E	✓	All NRT and NTC reactions for this study were ≤ 8% based on the following formula: RT+ fluorescence = 100% − [2^(−(Cq NRT or NTC)/(−2^(−Cq RT+))^ × 100] [37].
Standard curves with slope and y-intercept	E	✓	See Table 1 and Figure A1.
PCR efficiency calculated from slope	E	✓	See Table 1.
Confidence interval for PCR efficiency or standard error	D		
r2 of standard curve	E	✓	See Table 1.
Linear dynamic range	E	✓	See Table 1.
Cq variation at lower limit	E	✓	See Table 1.
Confidence intervals throughout range	D		
Evidence for limit of detection	E	✓	See Table 1.
If multiplex, efficiency and LOD of each assay.	E	✓	Not Multiplex
**Data Analysis**			
qPCR analysis program (source, version)	E	✓	BioRad Manager 3.1 software (updated 06/06/2021)
Cq method determination	E	✓	The values of Cqs were measured using the regression determination method of BioRad Manager 3.1 software (updated 06/06/2021).
Outlier identification and disposition	E	✓	Outliars included
Results of NTCs	E	✓	All NRT and NTC reactions for this study were ≤ 8% based on the following formula: RT+ fluorescence = 100% − [2^(−(Cq NRT or NTC)/(−2^(−Cq RT+))^ × 100] [37].
Justification of number and choice of reference genes	E	✓	Three reference genes were assessed due to a recommended number [38], but only the two most stable reference genes were used to calculate relative expression (Figure 2 and Figure 3).
Description of normalization method	E	✓	Relative quantification for mRNA expression levels [37]
Number and concordance of biological replicates	D	✓	Pooled cDNA (biological replicates 3–4). Figure 1.
Number and stage (RT or qPCR) of technical replicates	E	✓	Two technical replicates of tissue samples were performed from pooled cDNA
Repeatability (intra-assay variation)	E	✓	All but 3 technical replicates out of 210 conducted were within 0.5 Cqs.
Reproducibility (inter-assay variation, %CV)	D	✓	The percent coefficient of variation [(standard deviation/mean) × 100] of intraassay controls was below 1.3% (Figure A3).
Power analysis	D		
Statistical methods for result significance	E	✓	Data were analyzed by a Two-Way ANOVA with either a Sidak or Tukey multiple comparison test. Adjusted *p*-values ≤ 0.05 were considered statistically significant.
Software (source, version)	E	✓	All analyses were conducted using GraphPad Prism version 9.3.1 for Mac OS (GraphPad Software, San Diego, CA, USA).
Cq or raw data submission using RDML	D		

**Table A2:** Pooled sample specifics. The mean and standard deviation (SD) are reported for the RNA IQ, 260/280, and the total quantity of each cDNA pool.

Tissue	Species	IQ		260/280		Total RNA Extracted (μg)	
		Mean	SD	Mean	SD	Mean	SD
Brain	Steers	8.3	0.4	2.16	0.01	36	12
	Wethers	7.1	0.7	2.14	0.02	45	8
Duodenum	Steers	8.8	1.4	2.04	0.02	30	9
	Wethers	9.2	0.1	2.07	0.01	32	6
Jejunum	Steers	9.2	0.3	2.04	0.02	37	6
	Wethers	8.9	0.6	2.06	0.01	57	20
Ilium	Steers	9.2	0.5	2.11	0.10	83	9
	Wethers	9.3	0.6	2.07	0.07	80	4
Cecum	Steers	7.2	0.3	2.01	0.03	68	8
	Wethers	6.9	0.3	2.01	0.01	57	21
Colon	Steers	9.1	0.9	2.09	0.02	42	31
	Wethers	9.6	0.8	2.07	0.02	32	19
Rumen	Steers	8.6	1.0	2.10	0.02	35	16
	Wethers	8.5	1.2	2.10	0.04	40	13
Reticulum	Steers	9.4	0.5	2.17	0.03	23	6
	Wethers	9.4	0.1	2.20	0.06	23	9
Omasum	Steers	9.5	0.9	2.07	0.01	39	4
	Wethers	9.9	0.2	2.08	0.01	30	5
Abomasum	Steers	9.6	0.3	2.14	0.01	68	7
	Wethers	9.3	0.3	2.16	0.08	78	14
Muscle	Steers	8.7	0.9	2.10	0.01	11	1
	Wethers	8.5	1.1	2.10	0.01	7	2
Fat	Steers	7.5	0.6	1.95	0.08	5	4
	Wethers	8.1	0.5	1.96	0.08	7	3
Liver	Steers	9.1	0.5	2.10	0.01	33	22
	Wethers	8.0	0.4	2.15	0.01	25	9
Spleen	Steers	8.6	1.1	2.06	0.00	19	5
	Wethers	9.2	0.6	2.05	0.01	16	5
Kidney	Steers	9.8	0.4	2.08	0.02	49	12
	Wethers	8.9	0.1	2.08	0.03	45	2
